# Needs for family caregivers of Cerebrovascular Accident survivors

**DOI:** 10.17533/udea.iee.v38n3e06

**Published:** 2020-11-11

**Authors:** Jaine Kareny da Silva, Karla Ferraz Anjos, Jeorgia Pereira Alves, Darci de Oliveira Santa Rosa, Rita Narriman Silva de Oliveira Boery

**Affiliations:** Nurse, Ph.D. Professor, Universidade do Estado da Bahia, Guanambi-BA, Brazil. Email: jainekareny@yahoo.com.br Universidade do Estado da Bahia Universidade do Estado da Bahia Brazil jainekareny@yahoo.com.br; 2 Nurse, Ph.D. Universidade Federal da Bahia, Salvador-BA, Brazil. Email: karla.ferraz@hotmail.com Universidade Federal da Bahia Universidade Federal da Bahia Brazil karla.ferraz@hotmail.com; 3 Physiotherapist, Ph.D. Universidade Estadual do Sudoeste da Bahia, Jequié-BA, Brazil. Email: jeuaquino@gmail.com Universidade Estadual do Sudoeste da Bahia Universidade Estadual do Sudoeste da Bahia Brazil jeuaquino@gmail.com; 4 Nurse, Ph.D. Professor, Universidade Federal da Bahia, Salvador-BA, Brazil. Email: darcienf@ufba.br Universidade Federal da Bahia Universidade Federal da Bahia Brazil darcienf@ufba.br; 5 Nurse, Ph.D. Professor, Universidade Estadual do Sudoeste da Bahia, Jequié-Bahia, Brazil. Email: rboery@uesb.edu.br Universidade Estadual do Sudoeste da Bahia Universidade Estadual do Sudoeste da Bahia Brazil rboery@uesb.edu.br

**Keywords:** needs assessment, caregivers, family, stroke, homebound persons, survivors, qualitative research, evaluación de necesidades, cuidadores, familia, accidente cerebrovascular, personas impossibilitadas, sobrevivientes, investigación cualitativa, determinação de necessidades de cuidados de saúde, cuidadores, família, acidente vascular cerebral, pacientes domiciliares, sobreviventes, pesquisa qualitativa

## Abstract

**Objective.:**

To know the needs of family caregivers of Cerebrovascular Accident survivors.

**Methodology.:**

This is a qualitative, descriptive, and exploratory study. Data were collected from 37 family caregivers of Cerebrovascular Accident survivors from a city in the interior of Bahia, through an interview using a semi-structured form, between September 2017 and March 2018, and submitted to thematic content analysis.

**Results.:**

Three categories emerged: the early need for health education on the disease and care for family caregivers; the need to restructure care for family caregivers; 3) family caregivers need free time for social activities and (self)care.

**Conclusion.:**

Caregivers have basic needs for health care and social interaction, which can enable by educational health interventions.

## Resumen

**Objetivo.:**

Conocer las necesidades de los cuidadores familiares de sobrevivientes de accidente cerebrovascular.

**Metodología.:**

Estudio cualitativo, descriptivo y exploratorio. Los datos se recogieron de 37 cuidadores familiares de sobrevivientes de accidente cerebrovascular de una ciudad en el interior de Bahía, mediante una entrevista semiestructurada, entre septiembre de 2017 y marzo de 2018, y sometidos al análisis temático de contenido.

**Resultados.:**

Surgieron tres categorías: 1) la necesidad temprana de educación en salud sobre la enfermedad y la atención a los cuidadores familiares; 2) la necesidad de reestructurar la atención a los cuidadores familiares; 3) necesidad de tiempo libre para los cuidadores familiares para las actividades sociales y (auto)cuidado.

**Conclusión.:**

Se encontró que los cuidadores tienen necesidades básicas de atención en salud e interacción social, lo que puede ser posible gracias a las intervenciones educativas en salud.

## Resumo

**Objetivo.:**

Conhecer as necessidades de cuidadores familiares dos sobreviventes de Acidente Vascular Cerebral.

**Metodologia.:**

Estudo qualitativo, descritivo e exploratório. Os dados foram coletados com 37 cuidadores familiares dos sobreviventes de Acidente Vascular Cerebral de uma cidade no interior da Bahia, por meio de entrevista com uso de formulário semiestruturado, entre setembro de 2017 e março de 2018, e submetidos à análise de conteúdo temática.

**Resultados.:**

Emergiram três categorias: necessidade precoce de educação em saúde sobre a doença e cuidados à cuidadores familiares; necessidade de reestruturação da assistência à cuidadores familiares; necessidade de tempo livre aos cuidadores familiares para as atividades sociais e (auto)cuidado.

**Conclusão.:**

Verificou-se que os cuidadores possuem necessidades básicas de atenção à saúde e interação social, que podem ser viabilizadas por intervenções educativas em saúde.

## Introduction

Cerebrovascular Accident (CVA) is a neurological and degenerative disease with two different phases, the acute phase characterized by the patient's transition from hospital to home, and the chronic phase, which begins at six months post-stroke and lasts over time.(1) The limitation of these phases is important due to the varied biopsychosocial changes in the short and long term of the survivors of this disease and, concomitantly, the demands for specific care that sometimes need support from others such as family caregivers. Most stroke survivors have cognitive, psychological, and motor deficits that reduce their ability to perform basic daily activities and social participation,(2) making them dependent on their families to provide care during community rehabilitation. As a consequence, these caregivers experience the burden of care, highlighted by the deterioration of physical and mental health, which affects the worsening of their Quality of Life (QOL).(3)

These caregivers need to receive continuous support from a formal support network, from the moment of hospital admission to the return home, including assistance to their health and training for care.(4) However, it is not standard clinical practice for services, and health professionals offer this support to the caregivers. Studies point out that adequate social support must be preceded by the investigation of their needs,(5- 7,9) especially in the chronic phase of the CVA because the caregiver's burden is still critical in this phase, especially due to lack and/or incipience interventions in health.(10) To describe these needs, Bradshaw developed the concept of felt and expressed needs. The felt needs represent the individual desires and want under the perception of each person and the expressed needs are the needs of a person looking for a health service,(11) and they are strongly recommended by systematic reviews.(12,13) Considering this scenario, the objective of this study is to know the needs of family caregivers of Cerebrovascular Accident survivors.

## Methods

This is exploratory and descriptive research with a qualitative approach. It is part of a larger project entitled “Perception of health professionals and family caregivers about the care of people after a Cerebrovascular Accident”. In this, the needs of caregivers were investigated to subsidize the planning and construction of a social support intervention for families.

Family caregivers of Cerebrovascular Accident survivors participated in the study. The data collection occurred with saturation, which is a criterion commonly used in qualitative research to determine the sample from the moment when data collection no longer clarifies the object of study.(14) The inclusion criteria adopted were: being 18 years old or older, declaring to be the primary caregiver of a family member dependent on care after the stroke, living in an urban area during the production of the data, and presenting care time between six and 50 months (considered a chronic phase stroke). The choice of the chronic phase of the stroke was related to learning the long-term care needs of dependent people who have already gone through the acute often unexpected and (re) adaptation phase.

The selection of the participants was after the initial identification of CVA Accident survivor’s dependent on home care. Thus, we searched the medical records of a public hospital and the 17 Basic Health Units (UBS), located in the urban area of the city of Guanambi, in the interior of Bahia, for people with a medical diagnosis of a stroke. Subsequently, we scheduled a home visit with family caregivers to identify stroke survivors who were in the chronic phase of the disease and with functional dependence. In the health services records, we identified 14 people from the BHU and 23 from the hospital, totaling 37 people. All caregivers experienced the act of caring in both health units, taking care of patients in the chronic post-CVA phase, and were monitored by the UBS professionals.

To evaluate the functional dependence of CVA survivors, we used the Basic Activities of Daily Living (BADL) scale developed by Sidney Katz.(15) A specialist nurse trained by the main researcher applied this scale. It is an instrument that evaluated the functional independence of the BADL of the people being needing care, the stroke survivors, in six hierarchical functions (food, sphincter control, transference, condition of going to the bathroom, ability to dress and bathe). The score used ranges from zero to six points, in which one point is assigned to each “Yes” answer, with the individual being classified as independent in all functions (score zero) or dependent from one to six functions (score from one to six).(15)

The main researcher identified the caregivers' needs through interviews, using a semi-structured form in their respective homes, from September 2017 to March 2018. The research questions were: “What are your personal needs since becoming a caregiver of your relative? What are your needs related to the care of your family member since you became a caregiver?” We used an audio recorder for the testimonies that lasted an average of 30 minutes each. For the treatment of data, we used the technique of analysis of categorical thematic content, which consisted of three stages: 1) The pre-analysis that corresponds to a systematic organization of the data transcribed for reading, construction of the text corpus, and guiding the following analytical operations 2) The exploration of the material that promotes the conversion of the raw data collected into categories and/or subcategories by coding the units of analysis. 3) Finally, the treatment of the results, the inference, and the interpretation that involve apprehending the manifest and latent contents to the construction of the categories and/or subcategories by proposing inferences and interpretations based on them, interrelating them with the theoretical reference.(16)

All participants agreed to participate and signed the Informed Consent Form. The anonymity of family caregivers was guaranteed by the acronym "FC", followed by a different number for each of them, according to the sequence of their participation. The Research Ethics Committee of the State University of Southwest Bahia (UESB), campus Jequié approved this study with opinion 2,187,869 and CAAE 71341017.5.0000.0055.

## Results

Thirty-seven family caregivers participated in this study. The age of the caregivers ranged from 18 to 77 years old, 35 were female, 26 married, 25 had financial income below to one minimum wage, 23 did not work and 20 studied until complete elementary school, 23 lived with stroke survivors, 16 were children and 15 were spouses. Although all of them did not receive formal instruction for the role of caregiver, 15 of them used popular knowledge for their practice for more than 16 hours a week. In the analysis process, three empirical categories emerged: 1) early need for health education about the disease and care for family caregivers; 2) the need to restructure care to family caregivers; 3) need for free time for family caregivers for social activities and (self) care.

### The early need for health education about the disease and care for family caregivers

Although the caregivers interviewed take care of Cerebrovascular Accident survivors in the chronic phase, in this category emerged their needs to receive effective guidelines on the stroke and daily care since the acute phase of the stroke, as they were experienced moments that demanded formal support. This was expressed in the following statement: *At the beginning [from hospitalization to community return], I had doubts [...]. When the person has a stroke, we [family members] are very insecure about what will happen [...], so we need more guidance for day-to-day care* (FC03). Caregivers reveal that this deficit of guidelines listed in the context experienced comprises a gap in the knowledge of their praxis, marked by the difficulty in recognizing the risk factors, signs and symptoms of a stroke, and the possible recurrence of the disease. The participants reported these issues: *I don't know how to recognize the “stroke” [CVA] and I don't even know why he [husband] had this disease [...]* (FC21). *I don't know what the “stroke” [AVC] is like, what caused it and if it can be repeated* (FC11).

For caregivers who acquired knowledge about the disease on their initiative in research or from the experience of the incidence of stroke episodes, the main doubts were related to the recovery of the compromised functions, especially to physical disability and the exact cause of the stroke to prevent their recurrence. They stated and/or asked: *if we know what caused the stroke, we will fight [...]. In addition to physical therapy, what can be done to make the movements return?* (FC22). *What are the chances of the person returning to what was before [recovering] from the stroke?* (FC35). Regardless of the family caregivers´ age, they took care according to the knowledge they acquired during their daily lives and taking care of other people, sick or not. However, there are still several doubts related to the instrumentalization of basic daily care, such as food, locomotion and transfer, bathing, clothing, medications, seizure care, and treatment of pressure injuries, which should have already been addressed, considering the long-time of post-stroke care. Some of these issues are cited here: *I had doubts about picking him up, [...] moving him and lifting him [...] from one place to another [...]* (FC24). *Would a water mattress be better than an egg crate mattress to prevent injury? Is sunflower oil essential or is there a better one? [...]* (FC06). *I wanted someone to guide me to take care of her, [...], how to give a bath because I don't know if I take care of her correctly [...]. There are times when I hurt her and she is full of purple marks [bruises] [...]* (FC16).

### The need to restructure care to family caregivers

Caregivers inform that there is a delay in scheduling exams and consultations, lack of priority in care, failure in the referral and counter-referral system, and lack of home visits by the staff of the local health unit. These statements explain their difficulties: *Professionals should pay more attention to the person who cares. When I get to the “health center” [UBS], there should have more priority in the service* (FC25). [...] *The [community] health agent never came here again. [...] The nurse should come to our house [...] sometimes, to see how [we, caregivers] are doing, check the pressure [...], measure [the capillary blood glucose] with the tape [...]* (FC34). *“[...] I am not well received at the “health center” [UBS]. [...] When he gets sick, nobody [from the health team] comes [to the house]. I had to leave him [husband] lying in bed twice to get an exam. [...] Once, they [employees] forgot to schedule my name for the appointment, and the second time the doctor took time to attend me. I had to go back and make his lunch [...]* (FC33).

They also described the importance of psychological support to talk about their daily anxieties and gain knowledge about how to act in stressful situations from their intrapersonal (internal conflicts) and interpersonal (conflicts with the patient) relationship. They stated: *I wanted a psychologist to help the mind because sometimes it is good to talk about losing the person, we like [...], it is like a preparation* (FC07). *[...] taking care of him [husband] makes his head “heavy” [many duties]. [...] There are many concerns and changes at the same time. [...]. If I "get" [consult] a psychologist [...], I will get better. I will be calmer* (FC19). *I wanted a psychologist to talk about stress and anxiety. [...] Sometimes, I have conflicts with her for any reason* (FC11).

The need for surgical, clinical (general and specific), and nutritional care - to improve health through dietary (re) education and weight reduction - were also mentioned: *I need to have hernia surgery [...], which increased due to her weight* (FC29*). I need the physiotherapist due to pain [in the sacral region], because I stay down for a long time during her bath [mother], especially when I do her intimate hygiene. I [weighing 68 kilos] hold her entire weight [73 kilos]* (FC30). *I wanted to go to a nutritionist because I am pre-diabetic, I am overweight and I diet on my own* (FC32).

### Need for free time for family caregivers for social activities and (self) care

When assuming the responsibility of caregivers, they faced the difficulty of leaving home and resuming their autonomy completely due to the little support received. However, when they find a moment for themselves, they take the opportunity to go to church, visit friends and family or practice another pleasant activity. At the time of prayer, caregivers feel good, with spiritual comfort, and living in the social environment leaves them calm, relaxed, and free. They reported: *Going out, traveling, and taking a walk is good for me because I distract the mind [...]* (FC11). *Prayer comforts the person [...]* (FC20). *I feel very good about going to church because I feel alive and free. Hearing the word [of God] is always great. I like being in the middle of my family [...]* (FC23). *Young caregivers and young adults in particular are still eager to resume occupational activities. Work is recognized not only as a source of income but also as a moment of leisure and distraction: Before, I [...] worked sewing to help with the expenses of the house. I went out on a Saturday afternoon to go to my brother's house, visit a friend, or take my sewing. It was like having fun [...]* (FC07).

## Discussion

Family caregivers perceive their main needs related to the care of Cardiovascular Accident survivors and have some knowledge on how to seek to remedy them, but they face informal and formal support barriers. This demonstrates the need for health education actions that problematize these difficulties and boost the autonomy of these people to change their life context, so they concentrate on the care of the other, but not to the detriment of actions for self-care.

Prolonged daily care, permeated by learning experiences, can awaken the feeling of security about the disease and care practices. However, in this study, despite the long-time of care, some home caregivers still have health education needs about stroke and basic daily care, which are more frequent at the beginning of the disease.(7,17) Maybe this occurs due to the scarcity or inexistence of effective dialogues between health professionals and family caregivers at home, especially in primary health care, which is the reference in health education practices. In this sense, caregivers should be advised early, in the first stroke episode and in recurrent episodes, to avoid compromising the recovery of the stroke survivor and the quality of the caregiver's health.(18)

This education should not result in a mere transmission of information based on a technical model, which disregards the social context and previous knowledge of caregivers.(19) Professionals need to stimulate caregivers' curiosity in advance, encouraging them to reflect on the problems of their reality, awakening their critical thinking. When caregivers relate dialogued learning to their social context, there is a greater possibility of understanding the disease, its impacts, and elementary care for patients and, consequently, reducing possible errors during care praxis. With this understanding, the person can transform the way of caring for others and themselves.

For the caregivers in this study who acquired knowledge in the care trajectory, the questions and concerns were focused on the recovery of motor and cognitive functions, the treatment of skin lesions, and the care in convulsive crises. This shows the need to maintain educational practices during home care,(4) as other problems emerge in daily life, especially those linked to the aging process of the binomial caregiver and the person having care, the chronicity and incidence of a stroke. This context reaffirms the importance of establishing an investigative routine of these needs in health units, in particular, in more socially vulnerable groups. Other studies identified that caregivers wanted to share their experiences with their peers (other caregivers) and health professionals in support groups. This exchange of experience showed that it is possible to manage negative feelings(7,20) and that they do not show a lack of love, but a need for assistance. Although in this study, caregivers did not report the need to express their feelings with other caregivers, they expressed the desire to talk to a psychologist, which reinforces the need to offer this mental health care.

Thus, it is important to develop unique therapeutic projects in primary health care to expand mental health care and to create support groups for these caregivers. Although they can be mediated by psychologists, other professionals may be included such as nurses due to the extensive bond with the family. In the area of physical health, the main need was to reduce musculoskeletal pain, which originates from inadequate postures since the beginning of care or from worsening pre-existing injuries related to the role of caregiver.(21) The need for health care for the control of chronic diseases, the general clinical evaluation, and the change in lifestyle reaffirm that these caregivers continue to postpone self-care due to the improvement of the health of the person who has care, often abdicating their well-being to the detriment of caring others.

Therefore, the health professionals should reinforce the importance of self-care,(17) making care possible for caregivers' physical and emotional problems,(12) as they have little or no social support, which allows them to be absent of their function to take care of their health. The lack of priority in caring for the caregiver, especially those at risk of developing or worsening chronic diseases, can trigger short to long-term spending on health systems and social security, and therefore deserves attention from government sectors.

This difficulty in finding time for themselves that also emerged in this study, despite the care is more intense at the beginning,(7) it can last for months or years, affecting the feeling of well-being and reducing the chances of returning from activities work outside the home, especially in young or middle-aged adult caregivers.(22) An international survey carried out in Japan monitored family caregivers throughout care and found that most of them had difficulty finding free time to go out and relax, which resulted in the maintenance of overload of activities related to care. For this research, the limitation of free time occurred for cultural reasons, as the caregivers had a high sense of responsibility and believed that their substitutes would not adequately perform the same activities.(8) However, this study showed that this limitation may have been influenced by the lack of support from other family members, a common scenario in the Brazilian context also evidenced in other studies.(3,23)

The different cultural realities (Japan and Brazil) may have influenced this perception of social support since in the east the shared care among family members does not generate conflicts since their division is tacit and naturally accepted, while in the west the caregiver, in most cases, it is a single-family member, who does not always choose this function.(24) Despite the different contexts, national and international, the needs of family caregivers of Cardiovascular Accident survivors remain throughout the long-term care. The social support is mainly required in the emotional, assistance, and informational aspects and in the free time.(4,8,25)

As a limitation of this study we have testimonies from family caregivers of a specific context, taking care of people with a specific disease, so we need to expand this investigation in other geographical and care contexts to meet other caregiver needs who care for family members with other (co) morbidities. However, the study demonstrated the importance of (re) knowing and valuing the sensitive listening of the needs of these caregivers to plan specific educational practices and directed to the demands of the people who take care and have care from others.

## Conclusion

We observed that the family caregivers of Cardiovascular Accident survivors have basic health needs and social interaction, remedied by health interventions, based on educational practices, and valuing popular knowledge as a way of encouraging them to become active people and able to modify their reality. They also have unmet needs such as guidance, assistance, and time available for self-care, which has influenced their illness related to the care of others.

We expect that the identification of caregivers' needs will contribute to subsidize the planning, development, and implementation of projects directed to the demands of these people, improvement of assistance in health services, and the possibility of ensuring the right to priority in care, according to the singularities of the caregivers.

## References

[1] Costa VS, Silveira JCC, Clementino TCA, Borges LRDM, Melo LP (2016). Efeitos da terapia espelho na recuperação motora e funcional do membro superior com paresia pós-AVC: uma revisão sistemática. Fisioter. Pesqui..

[2] Bensenor IM, Goulart AC, Szwarcwald CL, Vieira MLFP, Malta DC, Lotufo PA (2015). Prevalence of stroke and associated disability in Brazil: National Health Survey - 2013. Arq. Neuropsiquiatr..

[3] Costa TF, Costa KNFM, Martins KP, Fernandes MGM, Brito SS (2015). Sobrecarga de cuidadores familiares de idosos com acidente vascular encefálico. Esc. Anna Nery Rev. Enferm..

[4] Cameron JI, Naglie G, Silver FL, Gignac MA (2013). Stroke family caregivers' support needs change across the care continuum: a qualitative study using the timing it right framework. Disabil. Rehabil..

[5] ay CB, Bierhals CCBK, Santos NOD, Mocellin D, Predebon ML, Dal Pizzol FLF (2018). Nursing home care educational intervention for family caregivers of older adults post stroke (SHARE): study protocol for a randomised trial. Trials.

[6] Fernandes CS, Ângelo M (2016). Cuidadores familiares: o que eles necessitam? Uma revisão integrativa. Rev. Esc. Enferm. USP.

[7] Tsai PC, Yip PK, Tai JJ, Lou ME (2015). Needs of family caregivers of stroke patients: a longitudinal study of caregivers’ perspectives. Patient Prefer. Adherence.

[8] Watanabe A, Fukuda M, Suzuk M, Kawaguchi T, Habata T, Akutsu T (2015). Factors Decreasing Caregiver Burden to Allow Patients with Cerebrovascular Disease to Continue in Long-term Home Care. J. Stroke Cerebrovasc. Dis..

[9] Pesantes MA, Brandt LR, Ipince AI, Miranda J, Diez-Canseco F (2017). An exploration into caring for a stroke-survivor in Lima, Peru: Emotional impact, stress factors, coping mechanisms and unmet needs of informal caregivers. eNeurologicalSci..

[10] Lutz BJ, Camicia M (2016). Supporting the Needs of Stroke Caregivers Across the Care Continuum. J. Clin. Outcomes Manag..

[11] Bradshaw JR (2013). A Taxonomy of Social Need. In: Cookson R, Sainsbury R, Gledinning G. Jonathan Bradshaw on Social Policy: Selected Writings 1972 - 2011.

[12] Bakas T, McCarthy M, Miller ET (2017). Update on the state of the evidence for stroke family caregiver and dyad interventions. Stroke.

[13] Silva JK, Anjos KF, Cruz VS, Boery RNSO, Santa Rosa DO, Boery EN (2018). Intervenções para cuidadores de sobreviventes de acidente vascular cerebral: revisão sistemática. Rev. Panam. Salud Pública.

[14] Minayo MCS (2017). Amostragem e saturação em pesquisa qualitativa: consensos e controvérsias. Rev. Pesq. Qualitativa.

[15] Lino VTS, Pereira SRM, Camacho LAB, Ribeiro ST, Buksman S (2008). Adaptação transcultural da Escala de Independência em Atividades da Vida Diária (Escala de Katz). Cad. Saúde Pública.

[16] Bardin L (2016). Análise de conteúdo.

[17] Cameron JL, Naglie G, Green TL, Gignac MA, Bayley M, Huijbregts (2015). A feasibility and pilot randomized controlled trial of the "Timing it Right Stroke Family Support Program". Clin. Rehabil..

[18] Landeiro MJL, Martins TV, Peres HHC (2016). Percepção dos enfermeiros sobre dificuldades e necessidades informacionais dos familiares cuidadores de Pessoa de Pendente. Texto & Contexto Enferm..

[19] Sevalho G (2018). O conceito de vulnerabilidade e a educação em saúde fundamentada em Paulo Freire. Interface Comun. Saúde Educ..

[20] Christensen ER, Golden SL, Gesell SB (2019). Perceived Benefits of Peer Support Groups for Stroke Survivors and Caregivers in Rural North Carolina. N. C. Med. J..

[21] Bakas T, Jessup NM, McLennon SM, Habermann B, Weaver MT, Morrison G (2016). Tracking patterns of needs during a telephone follow-up programme for family caregivers of persons with stroke. Disabil. Rehabil..

[22] Schulz CH, Godwin KM, Hersh GI, Hyde LK, Irabor JJ, Ostwald SK (2017). Return to work predictors of stroke survivors and their spousal caregivers. Work.

[23] Jesus ITS, Orlandi AAS, Zazzetta MS (2018). Sobrecarga, perfil e cuidado: cuidadores de idosos em vulnerabilidade Social. Rev. Bras. Geriatr. Gerontol..

[24] Faller JW, Zilly A, Alvarez AM, Marcon SS (2017). Filial care and the relationship with the elderly in families of different nationalities. Rev. Bras. Enferm..

[25] Li X, Xia X, Wang P, Zhang S, Liu M, Wang L (2017). Needs and rights awareness of stroke survivors and caregivers: a cross-sectional, single-centre questionnaire survey. BMJ Open.

